# Bacteremia due to *Sphingomonas paucimobilis* in pediatric and adult patients at a reference hospital in Peru 2020-2024

**DOI:** 10.17843/rpmesp.2026.431.14786

**Published:** 2026-03-31

**Authors:** Stalin Vilcarromero, Olguita del Águila, Mario Agramonte-Vilca, Norka Luarte-Saldaña

**Affiliations:** 1 Hospital Nacional Edgardo Rebagliati Martins, EsSalud. Lima, Peru; 2 Universidad San Antonio Abad del Cusco, Cusco, Peru.

**Keywords:** Bacteremia, Drug Resistance, Cross Infection, Central Venous Catheters, Peru

## Abstract

*Sphingomonas paucimobilis* is a ubiquitous bacterium of low virulence that can cause bacteremia, especially in immunocompromised patients. Given the absence of recent reports on this pathogen and its resistance profile in Peru, this series evaluated its clinical and microbiological characteristics in a reference hospital. Six cases confirmed by blood culture between January 2020 and September 2024 were analyzed (4 pediatric, 2 adults). All patients presented multiple risk factors or comorbidities, with the majority using a central venous catheter. The mortality observed in the series was 50%. Regarding sensitivity, two isolates showed resistance to third- and fourth-generation cephalosporins and ciprofloxacin, all being sensitive to aminoglycosides and another quinolone such as levofloxacin. *S. paucimobilis* represents an emerging pathogen, with variable resistance patterns that highlight the need for continuous surveillance and individualized therapeutic management guided by an antibiogram.

## INTRODUCTION

*Sphingomonas paucimobilis* is an uncommon, non-fermenting bacillus that has been isolated from various fluids and tissues, with blood being the most frequent site of isolation ^1^^,^^2^. Although generally considered to be of low virulence compared to other Enterobacteriaceae, it can cause serious infections, including septic shock, and fatal outcomes in immunocompromised patients ^1^^,^^3^^,^^4^.

Its emergence as a cause of bacteremia is an important finding, as there are no recent reports on its resistance profile in Peru ^1^^,^^3^^-^^5^. The objective of this case series is to describe the clinical characteristics and resistance pattern of *Sphingomonas paucimobilis* during 2020-2024 at the Edgardo Rebagliati Martins Hospital in Lima, Peru.

## CASE REPORT

This is a study of a case series conducted at the Hospital Nacional Edgardo Rebagliati Martins in Lima, Peru, between January 2020 and September 2024. The study population included pediatric or adult patients hospitalized in various services. All patients who met the definition of bacteremia by *S. paucimobilis* were included: 1) Isolation of one or more blood cultures positive for *S. paucimobilis* and 2) Presence of signs and symptoms compatible with sepsis or bloodstream infection. Exclusion Criteria/Contamination Discard: Single isolates in patients without a clinical presentation of infection or those cases categorized in the medical history as sample contamination by the treating medical team were excluded.

According to the care protocol, blood cultures are taken from all patients with suspected sepsis, using adult or pediatric bottles as appropriate. These bottles are then placed in the BACTEC 9120 automated system (Becton Dickinson Diagnostic Instrument Systems, Sparks, Maryland). The identification of Sphingomonas paucimobilis was performed using the VITEK 2 Compact automated system (bioMérieux), which has recognized specificity and sensitivity in the literature for non-fermenting Gram-negative bacilli. Additionally, typical colonial morphology and biochemical tests of positive oxidase and yellow pigment production were confirmed. Antimicrobial sensitivity was determined by the Kirby-Bauer method; and the determination of the minimum inhibitory concentration (MIC) was carried out using Wider panels (Dade Microscan Inc., West Sacramento, USA). All procedures and the interpretation of antimicrobial sensitivity were performed strictly based on the standards and breakpoints of the Clinical and Laboratory Standards Institute (CLSI 2009).

### Case 1

A 38-week male newborn, full-term, born by cesarean section due to myelomeningocele detected on prenatal ultrasound, with Apgar 9/9, adequate weight for gestational age, with a wall defect and a 4 x 3 cm lumbosacral spinal canal defect with a ruptured cystic lesion containing meninges and nerve roots with scant serous content on the superficial part of the defect, additionally with an altered coagulation profile under the diagnosis of ruptured myelomeningocele and probable sepsis; blood culture samples were collected, and empirical antibiotic coverage was initiated with gentamicin 13.5 mg every 24 h and vancomicine 40 mg every 12 h, respectively. The blood culture result reported *S. paucimobilis*. The patient entered the operating room for repair. Postoperatively, he was connected to a mechanical ventilator and presented hypoactivity and somnolence, which later subsided. He also presented with hypocalcemia, hypoproteinemia, hypoalbuminemia, and indirect hyperbilirubinemia. On the eighth day, with a reduction in inflammatory markers, antibiotic therapy was discontinued. He was discharged for follow-up by the neurology outpatient clinic (table 1).

**Table 1:** Clinical characteristics, risk factors, and outcome of six cases of *Sphingomonas paucimobilis* bacteremia (2020-2024).

	Pediatric				Adults	
Case	1	2	3	4	5	6
Age	0 years	11 years	2 months	2 years	87 years	42 years
Medical history	38-week newborn born by cesarean section due to myelomeningocele. Apgar score 9/9. Height: 50cm / Weight: 3,370 g / HC: 35.5 cm / CC: 33 cm	Spastic cerebral palsy. Severe chronic malnutrition/marasmus	Intestinal malrotation operated on the 5th day of life, abdominal-source sepsis with empirical treatment.	Cerebral hypotrophy. Global developmental delay	Hypertension, diabetes mellitus, heart failure, chronic kidney disease	Refractory systemic sclerosis with primary biliary cirrhosis and heart failure. Cyclophosphamide and IVIg.
Previous use of broad-spectrum antibiotics (prior 90 days)		Ceftriaxone	Ceftriaxone, Amikacin		Vancomycin, Ceftriaxone, Ceftazidime, Ertapenem	
Reason for admission	Myelomeningocele and suspected neonatal sepsis	Respiratory infection due to SARS-CoV-2	Chronic diarrhea and probable milk protein allergy		Heart failure exacerbated by cardiac arrhythmia	Severe sepsis due to spontaneous bacterial peritonitis
Extrinsic Risk Factors	1st Surgery: Myelomeningocele repair; 2nd Surgery: Ventriculoperitoneal shunt	Central venous catheter, nasogastric tube, urinary catheter	Central venous catheter	Central venous catheter	Long-term high-flow central venous catheter	Venous catheter for TPN
Antibiotic cycle 1	Ampicillin, Gentamicin	Ceftriaxone (6 days), Vancomycin (15 days)	Meropenem, Vancomycin	Ceftriaxone	Vancomycin, Meropenem	Cefepime, Vancomycin, Caspofungin
Antibiotic cycle 2	Vancomycin, Gentamicin	Meropenem, Colistin, Caspofungin	Meropenem, Colistin, Caspofungin	Vancomycin (6d), Cefepime, Caspofungin (6d)	Meropenem	Meropenem, Caspofungin (35 days)
Antibiotic cycle 3		Meropenem, Cotrimoxazole	Meropenem, Cotrimoxazole			Ciprofloxacin, Oral Vancomycin
Total hospital stay	23 days	66 days	55 days	11 days	18 days	81 days
Blood culture *Sphingomonas* spp.	1st day of hospitalization	Day 38	Day 8	Day 2	5th day of hospitalization	Day 63
Other isolates	No	*Staphylococcus hominis*	*Acinetobacter baumannii complex*, *Stenotrophomonas maltophilia*	No	No	*Pseudomonas aeruginosa*, *Candida parapsilosis*
Attributed symptoms	Irritability	Fever and respiratory symptoms	Fever	Fever, diarrhea, and abdominal distension	Sepsis and respiratory failure	Diarrea and abdominal distension
Final condition	Improved, discharge	Deceased	Deceased	Improved, discharge	Deceased	Improved, discharge

RN: newborn; HC: head circumference; AC: abdominal circumference; VPS: ventriculoperitoneal shunt; CVC: central venous catheter; TPN: total parenteral nutrition; IVIg: Intravenous immunoglobulin.

### Case 2

An 11-year-old girl with incomplete childhood vaccinations and a history of spastic infantile cerebral palsy, dorsal scoliosis, and severe chronic malnutrition/marasmus was hospitalized for SARS-CoV-2 infection and a presumed case of bacterial pneumonia that evolved into septic shock. After receiving oxygen support with a high-flow cannula and antibiotic therapy including meropenem, vancomycin, and caspofungin, she presented a favorable clinical evolution and progressive withdrawal of supplemental oxygen. In this context, she developed hospital-acquired pneumonia that evolved into sepsis; blood cultures were taken, and empirical intravenous antibiotic coverage was started with colistin 28 mg every 12 h, vancomycin 28 mg every 6 h, and trimethoprim/sulfamethoxazole at a dose of 75 mg of trimethoprim every 8 h. *S. paucimobilis* was isolated in blood cultures, and the central venous catheter was replaced. She showed a decrease in acute-phase reactants and remained afebrile; however, the respiratory distress evolved poorly, and she passed away (table 1).

### Case 3

A 2-month-old female infant, with a history of eutocic delivery at 38 weeks and a birth weight of 3560 g, and IIIB intestinal malrotation diagnosed on the fifth day of life, for which she underwent surgery and was discharged 10 days later. She evolved with poor weight gain and persistent diarrhea, for which she was re-hospitalized for dehydration, electrolyte imbalance, and severe malnutrition, and a right central venous catheter (CVC) was placed.

Studies were initiated for malabsorption syndrome, congenital enteropathy, and food intolerance. Enteral feeding was attempted with amino acid-based nutritional formulas and breastfeeding. On the tenth day of hospitalization, she presented with hypoactivity, weak sucking, and fever, and 24 hours later, shock, for which she was admitted to the intensive care unit for hemodynamic support and initiation of empirical antibiotic therapy with meropenem and vancomycin, after taking blood cultures; only the transcatheter blood sample was positive, with isolation of *S. paucimobilis*. The right CVC was removed on the fifth day, and a new left CVC was placed for nutritional support. Her evolution was poor, with persistent hypothermia, pulmonary congestion, and respiratory failure requiring mechanical ventilator support, neutropenia, and thrombocytopenia, abdominal distension without signs of intestinal occlusion, subfebrile temperature, and finally, a new isolation from a transcatheter blood culture of *Acinetobacter baumianii*. According to the resistance patterns in the antibiogram, antibiotic therapy was optimized by discontinuing vancomycin and adding cotrimoxazole. Prolonged stay in the critical unit; several unsuccessful attempts were made to wean her from the mechanical ventilator. Various isolated febrile peaks occurred, for which she maintained empirical coverage with antifungals (caspofungin) and even colistin, based on risk factors for other resistant germs. Two months after admission, the patient passed away (table 1).

### Case 4

A 2-year and 5-month-old male infant, with a history of hydrocephalus and global developmental delay. He presented with diarrhea with mucus and abdominal distension of approximately one day’s duration and came to the emergency department due to thermal elevations and paroxysms of tonic posture and clonic movements of the febrile seizure type. Support therapy, hydration, and empirical antibiotics with ceftriaxone were initiated. During his evolution, he remained febrile, with persistent hypokalemia, and difficult peripheral venous access was reported; hypoxemia and respiratory failure were added, for which he received low-flow oxygen and, shortly after, a high-flow nasal cannula. A right jugular central venous catheter was placed. Chest X-ray showed adequate catheter placement without signs of consolidation or pulmonary congestion; additionally, diuresis was preserved, and there were no signs of intestinal dysfunction or occlusion. Twenty-four hours later, fever peaks persisted, so blood cultures were repeated, and antibiotic therapy was rotated to cefepime and vancomycin. Urine culture was negative, and Polymerase Chain Reaction (PCR) for respiratory viruses (influenza, para-influenza, respiratory syncytial virus, and SARS-CoV-2) was negative. At 72 hours, *S. paucimobilis* was reported only in the transcatheter blood sample. Subsequently, he evolved with an adequate clinical response and progressive oxygen weaning. He also presented with leukopenia and thrombocytopenia secondary to the infection, requiring a platelet transfusion on one occasion. Ten days after being admitted, the patient was discharged with a favorable clinical evolution (table 1).

### Case 5

An 87-year-old male patient with a history of arterial hypertension, diabetes mellitus, heart failure, and chronic kidney disease on renal replacement therapy for 8 years, who has carried a long-term central venous catheter for three years. Two months before admission, he presented episodes of hypotension during hemodialysis sessions, for which blood cultures were taken and *Enterobacter cloacae* was isolated. He received empirical therapy with intravenous ceftazidime; due to persistent bacteremia, he was switched to ertapenem, with partially favorable evolution and without performing a central venous catheter replacement; subsequent controls had negative blood cultures. He was discharged, but two weeks later, he was readmitted due to decompensated heart failure and cardiac arrhythmia. During that hospitalization, he presented episodes of confusional syndrome and an increase in inflammatory markers, for which new blood cultures were obtained and empirical antibiotic coverage with meropenem and vancomycin was started. *S. paucimobilis* was isolated, and it was decided to maintain the empirical therapy. He evolved afebrile, with mild chest pain but no signs of ischemia; he also presented dyspnea associated with bilateral pleural effusion requiring low-flow oxygen support with a tendency toward arterial hypotension, for which new blood cultures were drawn and thoracentesis was performed, obtaining 900 cc. The blood cultures were negative; however, the evolution was poor, and he passed away (table 1).

### Case 6

A 42-year-old female patient with a history of progressive systemic sclerosis refractory to monthly treatment with immunoglobulins and cyclophosphamide. She has primary biliary cirrhosis treated with ursodeoxycholic acid and heart failure. She entered the emergency service due to severe sepsis secondary to spontaneous bacterial peritonitis in the context of tense ascites and chronic malnutrition. Percutaneous abdominal drainage was performed, and she was administered high-dose intravenous immunoglobulin and rituximab. She then developed fungemia associated with a parenteral nutrition catheter and bacteremia by *Pseudomonas aeruginosa*, which were treated successfully. In this context, she began episodes of persistent diarrhea with abdominal pain, for which she was treated empirically with intravenous ciprofloxacin and stool and blood cultures were taken, with isolation in the latter of *S. paucimobilis*. With clinical improvement and remission of the diarrhea, she was discharged.

## DISCUSSION

Our findings suggest that *S. paucimobilis* infection can be of both community and nosocomial origin. Although it has been identified as an emerging pathogen associated with hospital risk factors ^(1-3)^, the timing of isolation in some of our cases opens the possibility of community acquisition. International literature supports this duality; an Australian study found that 77% of 282 episodes of *S. paucimobilis* bacteremia were of community origin ^2^. In our study, the isolation of the germ was obtained within a range of 1 to 63 days of hospitalization. Case 1, a newborn, had isolation on day 2, suggesting an early-onset infection, probably hospital-acquired, but it could also be considered sample contamination. In other cases, such as case 4 (day 2 of hospitalization) and case 6 (day 1 of hospitalization), the very early isolation suggests probable community acquisition. A previous report in the Amazon region of Peru identified *Sphingomonas* spp. in water sources, which could be related to community acquisition ^5^.

The presence of multiple pre-existing risk factors in almost all patients, such as the use of central venous catheters (in 5 of the 6 cases), chronic diseases, and immunosuppression, highlights the vulnerability of these patients and the relevance of healthcare settings. The high morbidity and mortality observed, with 50% mortality in the cases with the most serious outcomes, contrasts with the low mortality generally described in the literature, which documents cases with a good prognosis ^6^^,^^7^. It is important to note that although *S. paucimobilis* bacteremia may not have been the direct cause of death in all cases, but rather a contributing factor to the critical state of patients with multiple comorbidities, it highlights the importance of considering this microorganism as a significant causal agent in vulnerable patients with comorbidities. The pattern of comorbidities aligns with the literature, which has described that most pediatric cases occur in premature patients or those with chronic diseases ^6^. Outbreaks of this pathogen have been reported in onco-hematology units, highlighting its nosocomial role in populations at risk ^4^.

*S. paucimobilis* carries genes that express beta-lactamases, and variable susceptibility patterns to third-generation cephalosporins have been reported, and less frequently, resistance to quinolones, aminoglycosides, and Trimethoprim-Sulfamethoxazole ^6^^,^^7^. Resistance to carbapenems is infrequent, and resistance mediated by carbapenemases is even rarer ^4^. Two decades ago, combination therapy with amikacin and ceftriaxone was recommended, but currently, it is considered that treatment should be guided according to sensitivity patterns on a case-by-case basis, considering quinolones or carbapenems as initial therapies for the most severe infections ^4^. Among our isolates, all were sensitive to aminoglycosides, levofloxacin, and cotrimoxazole. However, isolated resistance to ciprofloxacin was observed in two isolates, which were also resistant to 3rd and 4th generation cephalosporins and piperacillin/tazobactam (table 2), which differs from larger series that report, on the contrary, high sensitivity ^8^. This pattern can be compared to environmental findings in another study, where resistance to beta-lactams and ciprofloxacin is highly prevalent in *Sphingomonas* species isolated from drinking water ^9^.

**Table 2:** Minimum inhibitory concentration and interpretation reported for *Sphingomonas paucimobilis* isolates.

Antibiotics	Case 1		Case 2		Case 3		Case 4		Case 5		Case 6	
Aztreonam	>8	R	>8	R	>16	R	>16	R	>16	R	>16	R
Cefepime	>8	R	8	S	>16	R	>16	R	8	S	<=2	S
Cefotaxime	<=1	S	8	S	16	I	16	I	<=2	S	<=2	S
Ceftazidime	8	S	16	I	16	I	16	I	4	S	8	S
Ceftriaxone	N/R	-	N/R	-	>2	S	>2	S	<=1	S	<=1	S
Ciprofloxacin	<=1	S	>2	I	>1	I	>1	I	0.5	S	1	S
Amikacin	<=16	S	<=16	S	<=8	S	<=8	S	<=8	S	<=8	S
Imipenem	2	S	<=1	S	<=1	S	<=1	S	<=1	S	2	S
Levofloxacin	<=2	S	<=2	S	1	S	1	S	<=0.5	S	<=0.5	S
Meropenem	<=1	S	<=1	S	<=0.5	S	<=0.5	S	<=0.5	S	2	S
Piperacillin/Tazobactam	<=16	S	<=16	S	>64	R	64	I	<=16	S	<=16	S
Trimethoprim/Sulfamethoxazole	<=2/38	S	<=2/38	S	<=2/38	S	<=2/38	S	<=2/38	S	<=2/38	S

The reported isolates were obtained using the Kirby-Bauer method; the minimum inhibitory concentration (MIC) was determined using Wider panels (Dade Microscan Inc., West Sacramento, USA). Procedures were performed according to the Clinical and Laboratory Standards Institute (CLSI 2009).

Given that our series is limited, instead of prioritizing a specific regimen, empirical options could be considered based on the sensitivity patterns observed in our environment, always highlighting that definitive treatment must be guided by the antibiogram. From the point of view of surveillance and prevention of Healthcare-Associated Infections (HAI), it is essential to describe the frequency and characteristics of bacteremia associated with devices such as the central venous catheter (CVC). Peruvian national regulations require the surveillance of this type of infection ^10^. In this context, the significant resistance (33% or 2/6 isolates) to third- and fourth-generation cephalosporins in *S. paucimobilis*, a pathogen with a clear association with CVC use in our series, becomes crucial evidence for the importance of active HAI surveillance in our center. Our cases were also characterized by prolonged hospitalization, which exposes patients to various cycles of antibiotic therapy, including empirical use until definitive isolation is obtained. Therefore, the identification of local resistance patterns requires a continuous review of HAI prevention strategies ^11^^,^^12^. This case series contributes to profiling, at least in an initial manner, the challenge constituted by the prevention and control of infections by emerging pathogens with variable resistance.

In conclusion, *Sphingomonas paucimobilis* is an emerging pathogen in our environment that affects patients with risk factors. Although its origin can be both community and nosocomial, its serious manifestations and variable resistance patterns highlight the importance of individualized diagnosis and treatment, guided by the antibiogram. Given the emergence of *S. paucimobilis* in our hospital environment and the high vulnerability of the affected population, there is an imperative need to reinforce hospital microbiological surveillance. This includes proactive tracking of possible environmental reservoirs such as water systems or contaminated medical solutions and continuous monitoring of resistance profiles. Active surveillance is key to preventing future nosocomial outbreaks, especially in critical and onco-hematology units, where this pathogen has shown to be a significant risk.
