# Supplementary material for: Bacteremia due to Sphingomonas paucimobilis in pediatric and adult patients at a reference hospital in Peru 2020-2024
Source: Rev Peru Med Exp Salud Publica. 2026 Mar 31;43(1):132–7. doi: 10.17843/rpmesp.2026.431.14786 (PMC13245978; doi:10.17843/rpmesp.2026.431.14786)
Supplement: Translation

# Translation: Bacteriemia por *Sphingomonas paucimobilis* en pacientes pediátricos y adultos de un hospital de referencia del Perú 2020-2024

**Authors:** Stalin Vilcarromero, Olguita del Águila, Mario Agramonte-Vilca, Norka Luarte-Saldaña

**Affiliations:** 1 Hospital Nacional Edgardo Rebagliati Martins, EsSalud. Lima, Perú.; 2 Universidad San Antonio Abad del Cusco, Cusco, Perú.

**Keywords:** Bacteriemia, Resistencia a los Medicamentos, Infección Hospitalaria, Catéteres Venosos Centrales, Perú

## Abstract

*Sphingomonas paucimobilis* es una bacteria ubicua y de baja virulencia que puede causar bacteriemia, especialmente en pacientes inmunocomprometidos. Dada la ausencia de reportes recientes sobre este patógeno y su perfil de resistencia en Perú, esta serie evaluó sus características clínicas y microbiológicas en un hospital de referencia. Se analizaron 6 casos confirmados por hemocultivo entre enero de 2020 y septiembre de 2024 (4 pediátricos, 2 adultos). Todos los pacientes presentaron múltiples factores de riesgo o comorbilidades, con la mayoría utilizando catéter venoso central. La mortalidad observada en la serie fue del 50%. En cuanto a la sensibilidad, dos aislamientos mostraron resistencia a cefalosporinas de tercera y cuarta generación, y a ciprofloxacino, siendo todos sensibles a aminoglucósidos y a otra quinolona como el levofloxacino. *S. paucimobilis* representa un patógeno emergente, con patrones de resistencia variables que destacan la necesidad de una vigilancia continua y un manejo terapéutico individualizado guiado por antibiograma.

## INTRODUCCIÓN

*Sphingomonas paucimobilis* es un bacilo no fermentador, poco común, que se ha aislado en diversos fluidos y tejidos, siendo la sangre el sitio de aislamiento más frecuente ^1^^,^^2^. Aunque generalmente se considera de baja virulencia en comparación con otras enterobacterias, puede causar infecciones graves, incluyendo choque séptico, y desenlaces fatales en pacientes inmunocomprometidos ^1^^,^^3^^,^^4^.

Su emergencia como causante de bacteriemia es un hallazgo importante, ya que no existen reportes recientes sobre su perfil de resistencia en el Perú ^1^^,^^3^^-^^5^. El objetivo de esta serie de casos es describir las características clínicas y el patrón de resistencia de *Sphingomonas paucimobilis* durante el 2020-2024 en el Hospital Edgardo Rebagliati Martins, en Lima, Perú.

## REPORTE DE CASO

Estudio de una serie de casos, realizado en el Hospital Nacional Edgardo Rebagliati Martins en Lima, Perú, durante enero de 2020 y septiembre de 2024. La población de estudio incluyó pacientes pediátricos o adultos hospitalizados en diversos servicios. Se incluyeron todos los pacientes que cumplieron con la definición de bacteriemia por *S. paucimobilis*: 1) Aislamiento de uno o más hemocultivos positivos a *S. paucimobilis* y *2)* Presencia de signos y síntomas compatibles con sepsis o infección del torrente sanguíneo. Criterio de Exclusión/Descarte de Contaminación: Se excluyeron los aislamientos únicos en pacientes sin cuadro clínico de infección o aquellos casos catalogados en la historia clínica como contaminación de la muestra por el equipo médico tratante.

Según protocolo de atención, a todo paciente con sospecha de sepsis, se les toma de hemocultivo para adultos o pediátrico según corresponda. Estos frascos son luego colocados en el sistema automatizado BACTEC 9120 (Becton Dickinson Diagnostic Instrument Systems, Sparks, Maryland). La identificación de Sphingomonas paucimobilis se realizó mediante el sistema automatizado VITEK 2 Compact (bioMérieux), con una especificidad y sensibilidad reconocida en la literatura para bacilos Gram negativos no fermentadores. Además, se confirmó la morfología colonial típica y las pruebas bioquímicas de oxidasa positiva y producción de pigmento amarillo. La sensibilidad a antimicrobianos se determinó mediante el método de Kirby-Bauer; y la determinación de la concentración mínima inhibitoria (CMI), por medio de paneles de Wider (Dade Microscan Inc., West Sacrament, EE.UU.). Todos los procedimientos y la interpretación de la sensibilidad antimicrobiana se realizaron estrictamente en base a los estándares y puntos de corte del Clinical and Laboratory Standards Institute (CLSI 2009).

### Caso 1

Recién nacido de 38 semanas, género masculino, a término, nace mediante cesárea por mielomeningocele detectado en ecografía prenatal, con Apgar 9/9, peso adecuado para la edad gestacional, con defecto de pared y el canal raquídeo lumbosacro de 4 x 3 cm con lesión quística rota que contiene meninges y raíces nerviosas con escaso contenido seroso en la parte superficial del defecto, además con alteración del perfil de coagulación bajo el diagnóstico de mielomeningocele roto y sepsis probable, se recogen muestras para hemocultivos e inicia cobertura antibiótica empírica con gentamicina 13,5 mg cada 24 h y vancomicina 40 mg cada 12 h respectivamente. Se informó resultado de hemocultivo de *S. paucimobilis*. Ingresa a sala de operaciones para plastia. En el post operatorio sale acoplado a ventilador mecánico, presenta hipoactividad y somnolencia que luego remite. Cursa además con hipocalcemia, hipoproteinemia, hipoalbuminemia e hiperbilirrubinemia indirecta. Al octavo día, con reducción de marcadores inflamatorios se suspende la terapia antibiótica. Sale de alta para control por consultorio externo de neurología (tabla 1).

**Tabla 1:** Características clínicas, factores de riesgo y desenlace de seis casos de bacteriemia por *Sphingomonas paucimobilis* (2020-2024).

	Pediátrico				Adultos	
Caso	1	2	3	4	5	6
Edad	0 años	11 años	2 meses	2 años	87 años	42 años
Antecedente	RN de 38 semanas nacido por cesárea por mielomeningocele Apgar 9/9. Talla: 50 cm / peso: 3,370 g/ PC: 35,5 cm / CC: 33 cm	Parálisis cerebral infantil espástica Desnutrición crónica severa/marasmo	Malrotación intestinal operada al 5to día de vida, sepsis de foco abdominal con tratamiento empírico	Hipotrofia cerebral. retraso global del desarrollo.	Hipertensión arterial, diabetes mellitus, insuficiencia cardiaca, enfermedad renal crónica	Esclerosis sistémica refractaria con Cirrosis biliar primaria e insuficiencia cardíaca. Ciclofosfamida e IgEV
Uso previo de antibióticos de amplio espectro (90 días anteriores)		Ceftriaxona	Ceftriaxona Amikacina		Vancomicina Ceftriaxona Ceftazidima Ertapenem	
Motivo de ingreso	Mielomeningocele y sospecha de sepsis neonatal	Infección respiratoria por SARS-Cov2	Diarrea crónica y probable alergia a la proteína de la leche		Insuficiencia cardíaca exacerbada por arritmia cardíaca	Sepsis severa por peritonitis bacteriana espontánea
Factores de Riesgo extrínsecos	1ra. Cirugía: plastía de Mielomeningocele 2da. Cirugía: Derivación ventrículo peritoneal	Cateter venoso central, sonda nasogastrica, sonda vesical	Catéter venoso central	Catéter venoso central	Catéter Venoso de alto flujo de larga permanencia	Catéter Venoso para NPT
Ciclo antibiótico 1	Ampicilina Gentamicina	Ceftriaxona (6 días) Vancomicina (15 días)	Meropenem Vancomicina	Ceftriaxona	Vancomicina Meropenem	Cefepime Vancomicina Caspofungina
Ciclo antibiótico 2	Vancomicina Gentamicina	Meropenem Colistina Caspofungina	Meropenem Colistina Caspofungina	Vancomicina (6d) Cefepime Caspofungina (6d)	Meropenem	Meropenem Caspofungina (35 días)
Ciclo antibiótico 3		Meropenem Cotrimoxazol	Meropenem Cotrimoxazol			Ciprofloxacino Vancomicina Oral
Estancia hospitalaria total	23 días	66 días	55 días	11 días	18 días	81 días
Hemocultivo *Sphingomonas spp.*	1er día de hospitalización	día 38	al día 8	día 2	5to día de hospitalización	día 63
Otros aislamientos	No	*Stafilococcus hominis*	*Acinetobacter baumaniii complex Stenotrophomonas maltophilia*	No	No	*Pseudomonas aureginosa Candida parapsilosis*
Síntomas atribuidos	Irritabilidad	Fiebre y síntomas respiratorios	Fiebre	Fiebre Diarrea y distensión abdominal	Sepsis e insuficiencia respiratoria	Diarrea y distensión abdominal
Condición final	Mejorado, alta	Fallece	Fallece	Mejorado, alta	Fallece	Mejorado, alta

RN: recién nacido; PC: perímetro cefálico; PA: perímetro abdominal, DVP: derivación ventrículo-peritoneal; CVC: cateter venoso central, NPT: nutrición parenteral total; IgEv: Inmunoglobulina endovenosa

### Caso 2

Niña de 11 años con vacunas de infancia incompletas y antecedentes de parálisis cerebral infantil espástica, escoliosis dorsal y desnutrición crónica severa/marasmo es hospitalizado por infección por SARS CoV-2 y presunto cuadro de neumonía bacteriana que evoluciona a choque séptico. Luego de recibir soporte con oxígeno con cánula de alto flujo y terapia antibiótica con meropenem, vancomicina y caspofungina incluso, presenta evolución clínica favorable y retiro progresivo de oxígeno suplementario. En ese contexto, presenta neumonía intrahospitalaria que evoluciona a sepsis, se toman hemocultivos e inicia cobertura antibiótica empírica endovenosa con colistina 28 mg cada 12 h, vancomicina 28 mg cada 6 h y trimetoprim/sulfametoxazol a dosis 75 mg de trimetoprim cada 8 h. Se aísla *S. paucimobilis* en hemocultivos y se procede a cambiar el catéter venoso central. Presenta disminución de los reactantes de fase aguda y se mantiene afebril, sin embargo, la dificultad respiratoria evolucionó tórpidamente y fallece (tabla 1).

### Caso 3

Lactante de sexo femenino, de 2 meses de edad, con antecedentes de nacimiento por parto eutócico a las 38 semanas, con peso al nacer de 3560 g, malrotación intestinal IIIB diagnosticada al quinto día de vida por lo cual fue intervenida quirúrgicamente y fue dada de alta 10 días después, evolucionó con escasa ganancia ponderal y diarreas persistentes por lo cual es hospitalizada nuevamente por deshidratación, trastorno hidroelectrolítico y desnutrición severa y se instala catéter venoso central derecho (CVC).

Inicia estudios para síndrome de mala de malabsorción, enteropatía congénita e intolerancia alimentaria. Se ensaya alimentación enteral con fórmulas nutricionales a base de aminoácidos libres y lactancia materna. En el décimo día de hospitalización cursa con hipoactividad, succión débil y fiebre, y 24 horas después shock, por lo cual ingresa a la unidad de cuidados intensivos para soporte hemodinámico e inicio de terapia antibiótica empírica con meropenem y vancomicina, previa toma de hemocultivos y resultando positivo únicamente la muestra de sangre transcatéter, con aislamiento de *S. paucimobilis*. Se retira CVC derecho al quinto día, y se coloca nuevo CVC izquierdo para soporte nutricional. Su evolución es tórpida con hipotermia persistente, congestión pulmonar e insuficiencia respiratoria con soporte de ventilador mecánico, neutropenia y trombocitopenia, distensión abdominal sin signos de oclusión intestinal, subfebril y finalmente nuevo aislamiento de cultivo de sangre transcatéter con *Acinetobacter baumianii*. Según los patrones de resistencia en antibiograma se optimiza la terapia antibiótica, suspendiendo vancomicina y agregando cotrimoxazol. Estancia prolongada en unidad crítica, se intentó separar de ventilador mecánico en varias oportunidades sin éxito. Diversos picos febriles aislados por lo cual mantuvo cobertura empírica con antifúngicos (caspofungina) y colistina incluso, basado en los factores de riesgo para otros gérmenes resistentes. Dos meses después de su ingreso el paciente fallece (tabla 1).

### Caso 4

Infante masculino de 2 años y 5 meses de edad, con antecedentes de hidrocefalia y retraso global del desarrollo. Cursa con diarreas con moco y distensión abdominal de aproximadamente un día de evolución y que acude a emergencias por alzas térmicas y paroxismo de postura tónica y movimientos clónicos tipo convulsiones febriles. Inicia terapia de soporte, hidratación con antibióticos empírica con ceftriaxona. En su evolución persiste febril, con hipokalemia persistente y reportan acceso venoso periférico dificultoso; agrega hipoxemia e insuficiencia respiratoria por lo cual recibe oxígeno a bajo flujo y poco después cánula nasal de alto flujo. Se instala catéter venoso central yugular derecho. Radiografía de tórax con adecuada ubicación de catéter y sin signos de consolidación o congestión pulmonar, además diuresis conservada, no signos de disfunción u oclusión intestinal. Veinticuatro horas después persisten los picos de fiebre por lo que se repiten hemocultivos, y se rota terapia antibiótica a cefepime y vancomicina. Urocultivo negativo y Reacción en Cadena de la Polimerasa (PCR) para virus respiratorios (influenza, para-influenza, virus sincitial respiratorio y SARS-CoV2) negativos. Se reporta, a las 72 horas, *S paucimobilis,* únicamente en la muestra de sangre transcatéter. Posteriormente evolucionó con respuesta clínica adecuada y destete progresivo de oxígeno. Cursó además con leucopenia y trombocitopenia secundarias a la infección por lo cual requirió transfusión de plaquetas en una ocasión. Diez días después de ser admitido, paciente fue dado de alta con evolución clínica favorable (tabla 1).

### Caso 5

Paciente masculino de 87 años, con antecedentes de hipertensión arterial, diabetes mellitus, insuficiencia cardiaca y enfermedad renal crónica en terapia de reemplazo renal desde hace 8 años y porta catéter venoso central de larga permanencia desde hace tres años. Dos meses antes del ingreso, presenta episodios de hipotensión durante las sesiones de hemodiálisis, por lo cual, le toman hemocultivos y se aísla *Enterobacter cloacae*. Recibe terapia empírica con ceftazidima endovenosa, por persistencia de bacteremia cambia a ertapenem, con evolución parcialmente favorable y sin realizar recambio de catéter venoso central tuvo controles posteriores con hemocultivos negativos. Es dado de alta, pero dos semanas después reingresa por cuadro de insuficiencia cardiaca descompensada y arritmia cardiaca. En esa hospitalización, presenta episodios de síndrome confusional e incremento de marcadores inflamatorios por lo cual se obtienen nuevos hemocultivos e inicia empíricamente cobertura antibiótica con meropenem y vancomicina. Se aísla *S. paucimobilis*, se decide mantener la terapia empírica. Evoluciona afebril, con leve dolor torácico, pero sin signos de isquemia, además presenta disnea asociada a efusión pleural bilateral con apoyo de oxígeno a bajo flujo con tendencia a la hipotensión arterial, por lo cual se extraen nuevos hemocultivos y se realiza toracocentesis obteniéndose 900 cc. Los hemocultivos fueron negativos, sin embargo, la evolución es tórpida y fallece (tabla 1).

### Caso 6

Paciente mujer de 42 años, con antecedente de esclerosis sistémica progresiva refractario al tratamiento mensual con inmunoglobulinas y ciclofosfamida. Tiene cirrosis biliar primaria tratada con ácido ursodeoxicólico e insuficiencia cardíaca. Ingresa al servicio de emergencia por sepsis severa secundaria a peritonitis bacteriana espontánea en el contexto de ascitis a tensión y desnutrición crónica. Se le realiza drenaje percutáneo abdominal, le administran inmunoglobulina endovenosa a alta dosis y rituximab. Cursa entonces con fungemia *asociada* a catéter de nutrición parenteral y bacteriemia por *Pseudomona aeruginosa* los cuales son tratados exitosamente. En ese contexto, inicia episodios de diarreas persistentes con dolor abdominal por lo que fue tratado empíricamente con ciprofloxacino endovenoso y toma de coprocultivos y hemocultivos, con aislamiento en este último de *S. paucimobilis*. Con mejoría clínica y la remisión de la diarrea es dado de alta.

## DISCUSIÓN

Nuestros hallazgos sugieren que la infección por *S. paucimobilis* puede ser de origen comunitario como nosocomial. Aunque se ha identificado como un patógeno emergente asociado a factores de riesgo hospitalarios ^1^^-^^3^, el momento del aislamiento en algunos de nuestros casos abre la posibilidad de una adquisición comunitaria. La literatura internacional apoya esta dualidad; un estudio australiano encontró que el 77% de 282 episodios de bacteriemia por *S. paucimobilis* fueron de origen comunitario ^2^. En nuestro estudio, el aislamiento del germen se obtuvo en un rango de 1 a 63 días de hospitalización. El caso 1, un recién nacido, tuvo un aislamiento en el día 2, lo que sugiere una infección de inicio temprano, probablemente adquirida en el hospital, pero también podría considerarse una contaminación de la muestra. En otros casos, como el caso 4 (día 2 de hospitalización) y el caso 6 (día 1 de hospitalización), el aislamiento muy temprano sugiere una probable adquisición comunitaria. Un reporte previo en la región amazónica de Perú identificó *Sphingomonas* spp. en fuentes de agua, lo que podría estar relacionado con la adquisición en la comunidad ^5^.

La presencia de múltiples factores de riesgo preexistentes en casi todos los pacientes, como el uso de catéteres venosos centrales (en 5 de los 6 casos), enfermedades crónicas e inmunosupresión, resalta la vulnerabilidad de estos pacientes y la relevancia de los entornos de atención médica. La alta morbimortalidad observada, con un 50% de mortalidad en los casos de desenlace más grave, contrasta con la baja mortalidad generalmente descrita en la literatura, la cual documenta casos con buen pronóstico ^6^^,^^7^. Es importante notar que, si bien la bacteriemia por *S. paucimobilis* puede no haber sido la causa directa de la muerte en todos los casos, sino un factor contribuyente al estado crítico de pacientes con múltiples comorbilidades, resalta la importancia de considerar a este microorganismo como un agente causal significativo en pacientes vulnerables y con comorbilidades. El patrón de comorbilidades se alinea con la literatura, que ha descrito que la mayoría de los casos pediátricos ocurren en pacientes prematuros o con enfermedades crónicas ^6^. Los brotes de este patógeno se han reportado en unidades onco-hematológicas, destacando su rol nosocomial en poblaciones de riesgo ^4^.

*S. paucimobilis* porta genes que expresan betalactamasas y se han reportado patrones de susceptibilidad variable a cefalosporinas de tercera generación, y en menor frecuencia resistencia a quinolonas, aminoglucósidos y Trimetoprim-Sulfametoxazol ^6^^,^^7^. Es infrecuente la resistencia a carbapenémicos y aún más infrecuente la resistencia mediada por carbapenemasas ^4^. Hace dos décadas se recomendaba terapia combinada con amikacina y ceftriaxona pero actualmente se considera que el tratamiento debe guiarse según los patrones de sensibilidad caso por caso, considerando quinolonas o carbapenémicos como terapias iniciales para las infecciones más severas ^4^. Entre nuestros aislamientos, todos fueron sensibles a aminoglucósidos, levofloxacino y cotrimoxazol. No obstante, se observó una resistencia aislada al ciprofloxacino en dos aislamientos, los cuales fueron resistentes también a cefalosporinas de 3ra y 4ta generación y piperacilina/tazobactam (tabla 2), lo que difiere de otras más grandes, que reportan al contrario, alta sensibilidad ^8^. Este patrón se puede comparar a los hallazgos ambientales realizados en otro estudio, donde la resistencia a betalactámicos y ciprofloxacino es altamente prevalente en especies de *Sphingomonas* aisladas del agua potable ^9^. Dado que nuestra serie es limitada, en lugar de priorizar un régimen específico, las opciones empíricas podrían considerarse en base a los patrones de sensibilidad observados en nuestro medio, siempre destacando que el tratamiento definitivo debe ser guiado por el antibiograma.

**Tabla 2:** Concentración mínima inhibitoria e interpretación reportados para los aislamientos de *Sphingomonas paucimobilis.*

Antibióticos	Caso 1		Caso 2		Caso 3		Caso 4		Caso 5		Caso 6	
Aztreonam	>8	R	>8	R	>16	R	>16	R	>16	R	>16	R
Cefepima	>8	R	8	S	>16	R	>16	R	8	S	<=2	S
Cefotaxima	<=1	S	8	S	16	I	16	I	<=2	S	<=2	S
Ceftazidima	8	S	16	I	16	I	16	I	4	S	8	S
Ceftriaxona	N/R	-	N/R	-	>2	S	>2	S	<=1	S	<=1	S
Ciprofloxacino	<=1	S	>2	I	>1	I	>1	I	0.5	S	1	S
Amikacina	<=16	S	<=16	S	<=8	S	<=8	S	<=8	S	<=8	S
Imipenem	2	S	<=1	S	<=1	S	<=1	S	<=1	S	2	S
Levofloxacino	<=2	S	<=2	S	1	S	1	S	<=0.5	S	<=0.5	S
Meropenem	<=1	S	<=1	S	<=0.5	S	<=0.5	S	<=0.5	S	2	S
Piperacilina Tazobactam	<=16	S	<=16	S	>64	R	64	I	<=16	S	<=16	S
Sulfametoxazol Trimetoprim	<=2/38	S	<=2/38	S	<=2/38	S	<=2/38	S	<=2/38	S	<=2/38	S

Los aislamientos reportados fueron obtenidos mediante el método de Kirby-Bauer; la concentración mínima inhibitoria (CMI) se determinó por medio de paneles de Wider (Dade Microscan Inc., West Sacrament, EE.UU.). Los procedimientos se realizaron según Clinical and Laboratory Standards Institute (CLSI 2009).

Desde el punto de vista de la vigilancia y prevención de las Infecciones Asociadas a la Atención de Salud (IAAS), es fundamental describir la frecuencia y las características de las bacteriemias asociadas a dispositivos como el catéter venoso central (CVC). La normativa nacional peruana exige la vigilancia de este tipo de infecciones ^10^. En este contexto, la resistencia significativa (33% o 2/6 aislamientos) a cefalosporinas de tercera y cuarta generación en *S. paucimobilis*, un patógeno con una clara asociación al uso de CVC en nuestra serie, se convierte en una evidencia crucial para la importancia de la vigilancia activa de las IAAS en nuestro centro. Nuestros casos también se caracterizaron por una hospitalización prolongada, lo que expone a los pacientes a diversos ciclos de terapia antibiótica, incluyendo el uso empírico hasta obtener el aislamiento definitivo. Por ello, la identificación de patrones de resistencia local exige una revisión continua de las estrategias de prevención de IAAS ^11^^,^^12^. Esta serie de casos contribuye a perfilar, al menos de manera inicial, el reto que constituye la prevención y el control de infecciones por patógenos emergentes y con resistencia variable.

En conclusión, *Sphingomonas paucimobilis* es un patógeno emergente en nuestro medio que afecta a pacientes con factores de riesgo. Aunque su origen puede ser tanto comunitario como nosocomial, sus manifestaciones graves y patrones de resistencia variables resaltan la importancia del diagnóstico y tratamiento individualizado, guiado por el antibiograma.

Dada la emergencia de *S. paucimobilis* en nuestro entorno hospitalario y la alta vulnerabilidad de la población afectada, es imperativa la necesidad de reforzar la vigilancia microbiológica hospitalaria. Esto incluye el rastreo proactivo de posibles reservorios ambientales como sistemas de agua o soluciones médicas contaminadas y la monitorización continua de los perfiles de resistencia. Una vigilancia activa es clave para prevenir futuros brotes nosocomiales, especialmente en unidades críticas y onco-hematológicas, donde este patógeno ha mostrado ser un riesgo significativo.
